# Supplementary material for: A light-driven artificial flytrap
Source: Nat Commun. 2017 May 23;8:15546. doi: 10.1038/ncomms15546 (PMC5457518; doi:10.1038/ncomms15546)
Supplement: Supplementary Information — Supplementary Figures and Supplementary Notes [file ncomms15546-s1.pdf]

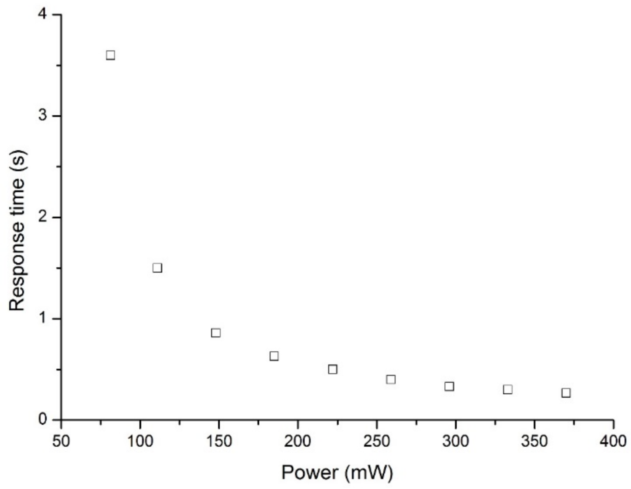

Supplementary Figure 1|. Closure speed vs. light power. A 4 mm sized flytrap gripper closes at a scattering cubic target driven by different laser powers. The response time is defined as the time between starting the irradiation and the moment when the gripper reaches the closure stage.

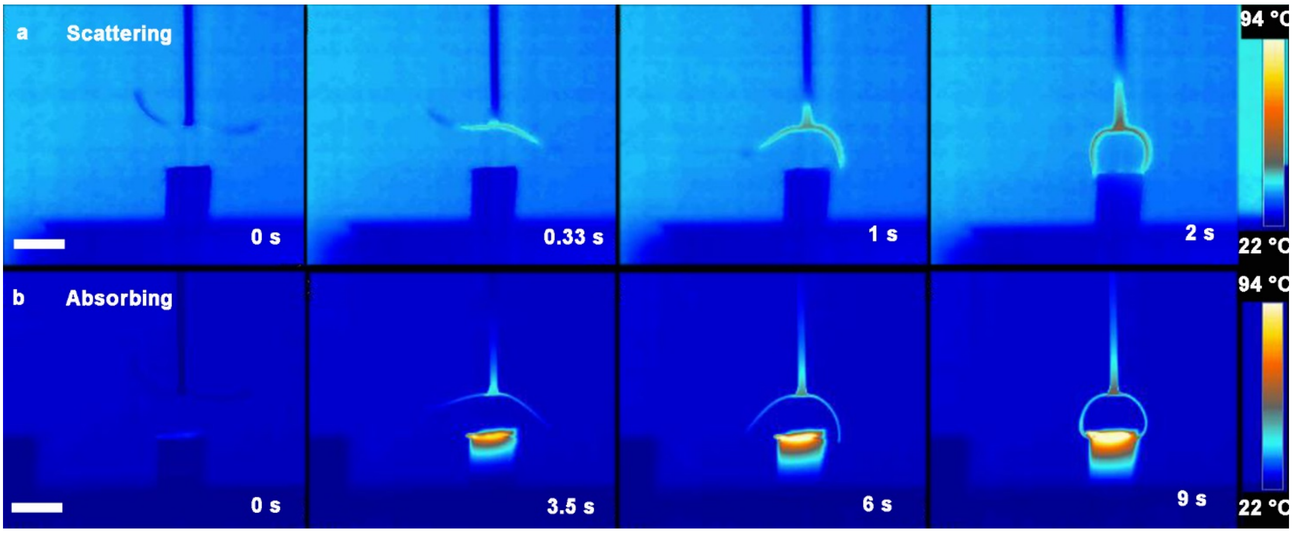

Supplementary Figure 2|. Thermal image of optical flytrap. Flytrap gripper at difference stages in front of scattering (**a**) and absorbing (**b**) targets. Laser power P = 94 mW. Scale bar: 2 mm.

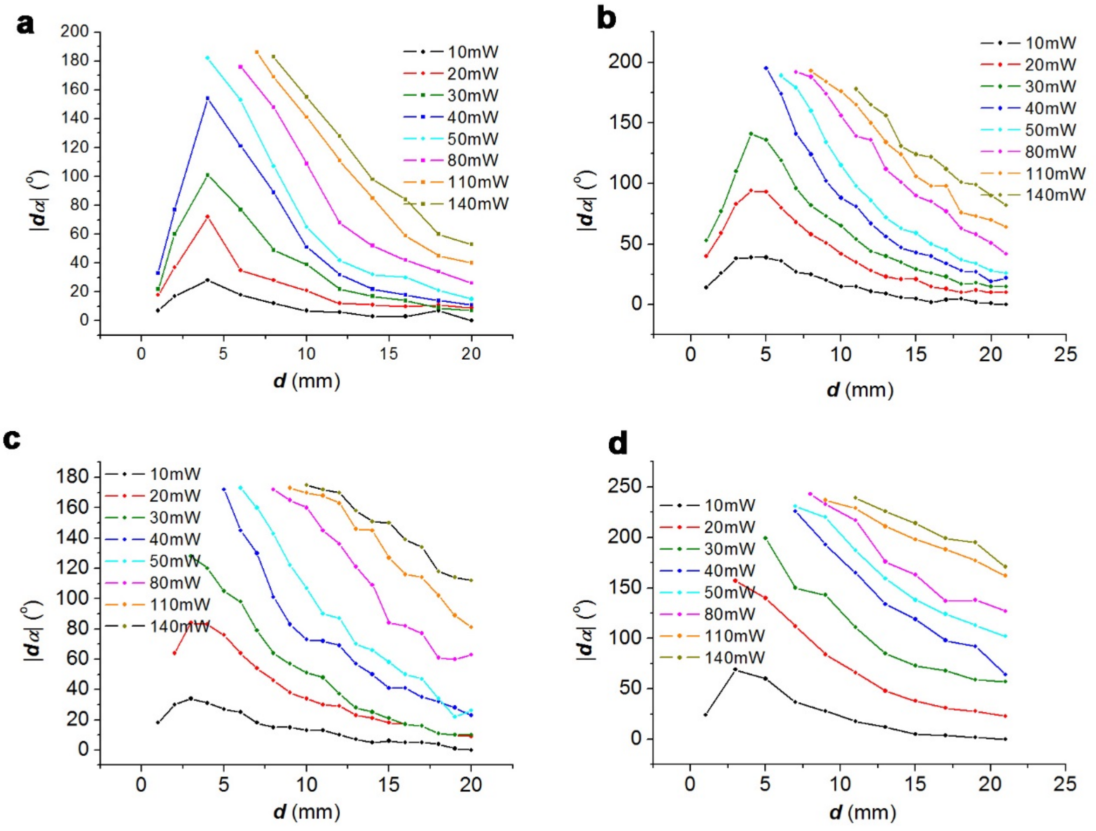

Supplementary Figure 3|. Size-dependent closure behavior in front of a mirror. Change of gripper angle $|d\alpha|$ as a function of distance d to the mirror surface at different light powers P. The LCE actuators used are 4 (**a**), 6 (**b**), 8 (**c**) and 10 (**d**) mm in length, 1 mm in width, and 20 μm in thickness.

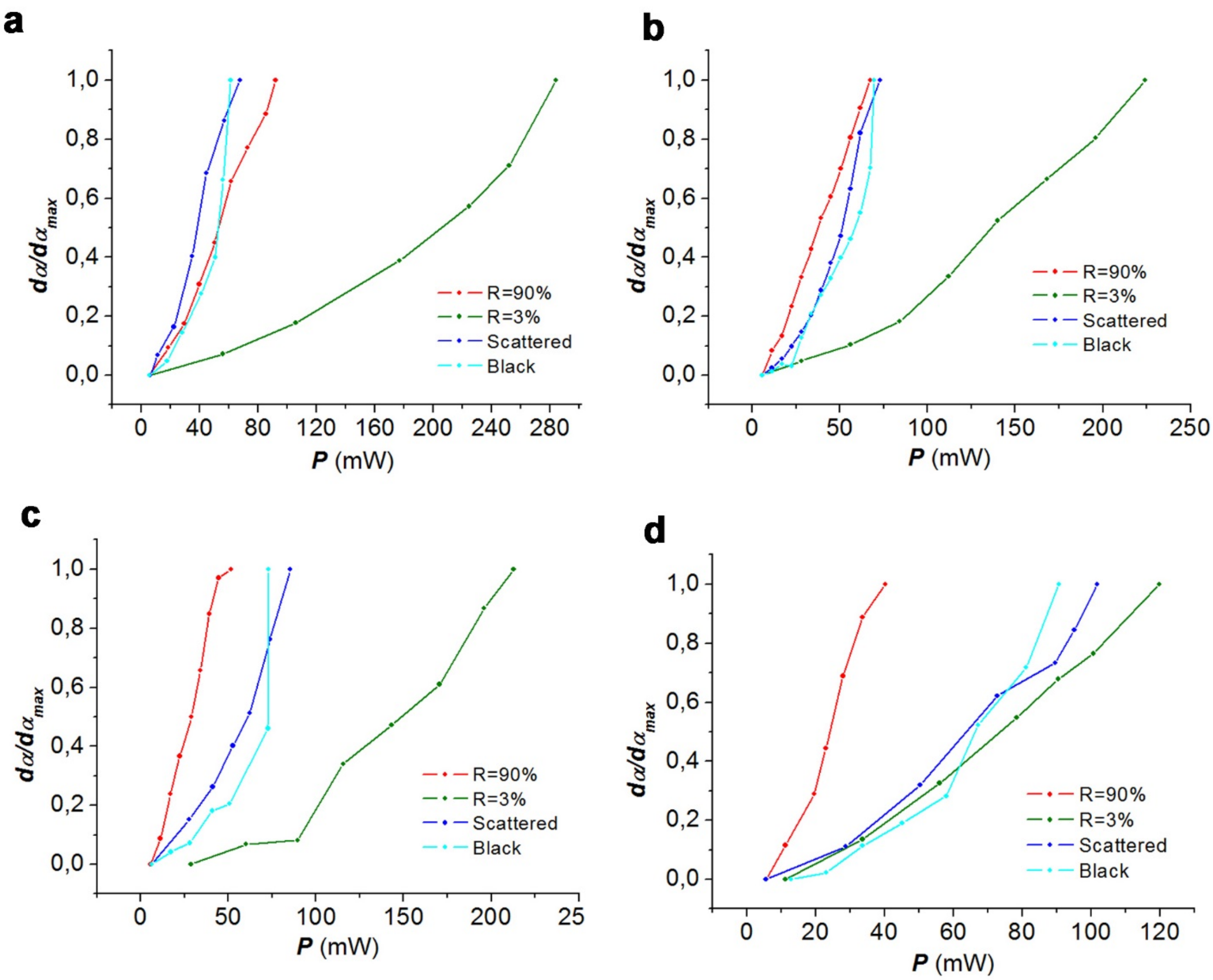

Supplementary Figure 4|. Self-recognition properties in different sized flytrap. Bending ratio $d\alpha/ d\alpha_{max}$ as a function of light power P in front of high-reflectivity (R: 90%), low-reflectivity (R: 3%), black (R: < 1%) and highly scattering targets. The LCE actuators used are 4 (**a**), 6 (**b**), 8 (**c**) and 10 (**d**) mm in length, 1 mm in width, and 20 μm in thickness.

Supplementary Note 1: Order parameter.

In order to measure the order parameter of the polymeric network, a uniaxially aligned liquid crystalline elastomer (LCE) thin film was prepared using the same polymerization conditions as for the splay-aligned film used for preparing flytrap, and its absorption spectra parallel and perpendicular to the molecular director were measured. The LC cell was formed by using PVA-coated glass slides (rubbed in the same direction) with a separation distance of 5 μm, followed by infiltration of the same LC monomer mixture, polymerized in an identical manner as explained in the Methods Section. The polarized absorption spectra are shown in **Supplementary Figure 5**.

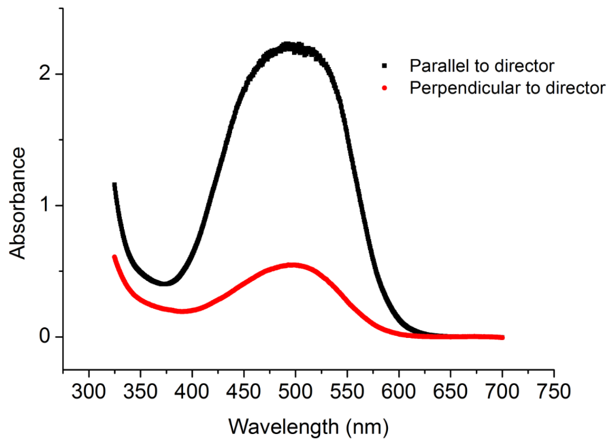

Supplementary Figure 5. Polarized absorption spectra of a homogeneously aligned LCE.

The order parameter, S_p, can be calculated as

$$S_p = \frac{A_{/\!/} - A_{\perp}}{A_{/\!/} + 2A_{\perp}}$$

where $A_{/\!/}$ and $A_{\perp}$ are the measured absorbance values with light polarized parallel and perpendicular to the LC alignment, respectively. S_p is determined to be 0.59 for the homogeneously aligned sample, by averaging the data in wavelength range 525 – 575 nm.

Supplementary Note 2: Bending-angle calculation.

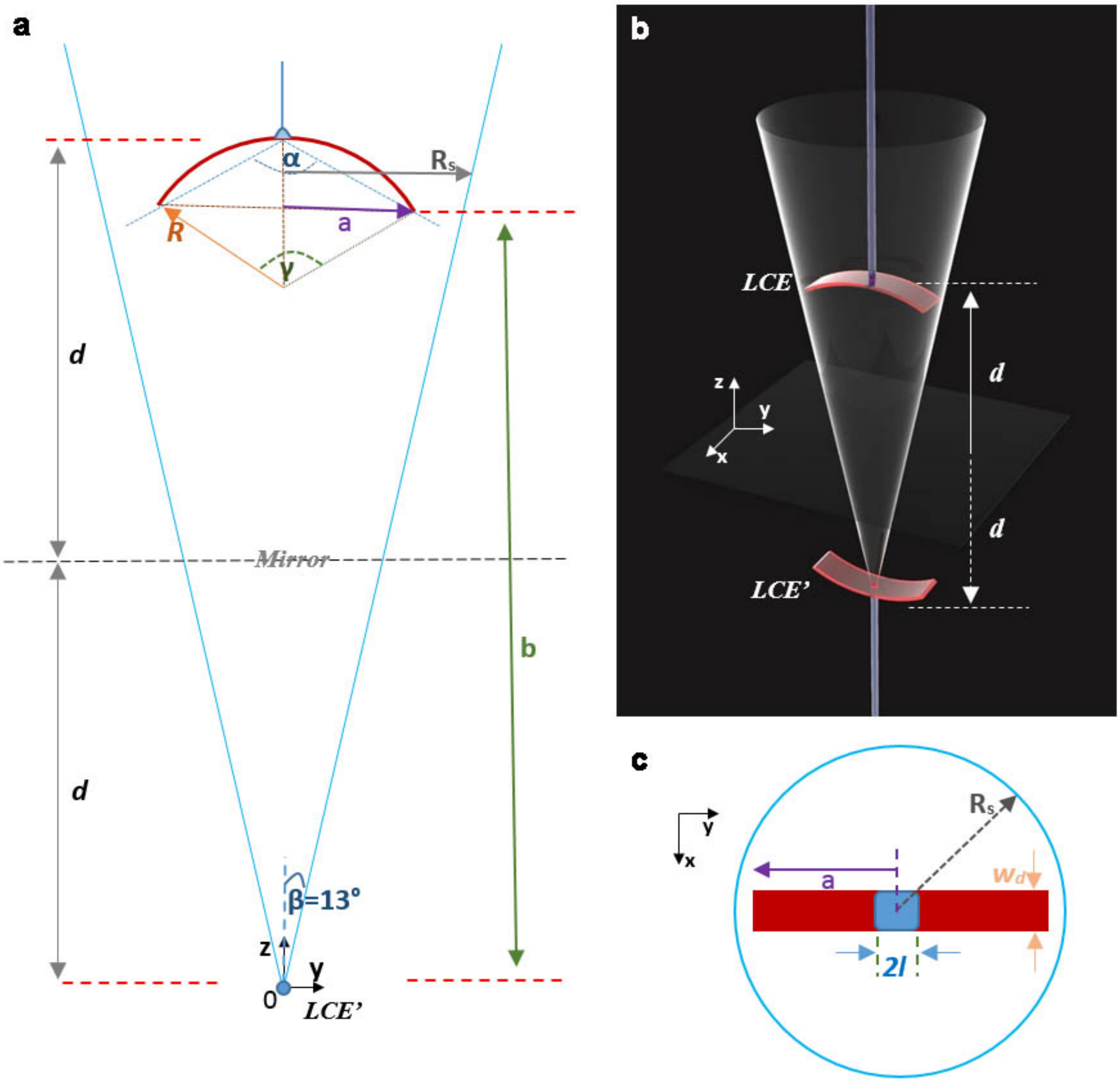

Supplementary Figure 6. Schematic drawing of light-induced deformation in the optical flytrap geometry.

The deformed gripper can be approximated as an arc with a central angle of γ and a curvature of $1/R$, as shown in **Supplementary Figure 6 (a)**. The relation between the measured gripping angle $\boldsymbol{\alpha}$ and the central angle γ is

$$tan\left(\frac{\alpha}{2}\right) = \frac{sin(\frac{\gamma}{2})}{1-cos(\frac{\gamma}{2})}. \qquad (1)$$

The change of central angle $d\gamma$ at an equilibrium stage depends on the absorbed light power E of the LCE stripe:

$$d\gamma = k \cdot E, \qquad (2)$$

where k is the light-induced bending coefficient ($k = 0.8$ rad·mW^{-1} for a 6 mm long LCE stripe). The key of the calculation is to find out the specific form of $E(P, d, \gamma)$, which depends on light power P, distance d and the deformed geometry (γ). For constant P and d, the LCE deforms into a well-defined, stable state. The central angle γ can be obtained by solving the equation

$$\gamma - \gamma_0 - k{\cdot}E(\gamma) = 0, \qquad (3)$$

where γ_0 is the original central angle of a 6 mm long LCE, $\gamma_0 = -112°$, $\alpha_0 = 236°$ (a negative value of γ_0, or say, $\alpha_0 > 180°$ is due to the inner stress in the photo-polymerized LCE film).

For a general case, E can be obtained using a surface integral

$$E = A \cdot P \cdot R \cdot \iint_S D(\theta,\varphi)\frac{\vec{\mathbf{r}}d\vec{\mathbf{S}}}{r^3} \qquad (4)$$

where $D(\theta, \varphi)$ is light density indicating the percentage of reflected light in $d\Omega = sin\theta d\theta d\varphi$ solid angle in spherical coordinates $<r, \theta, \varphi>$ ($\oint D(\theta,\varphi) \cdot sin\,(\theta) d\theta d\varphi = 1$), where R is the reflectivity of the surface, S is the area of actuated region of the LCE, and A is the absorbance of the LCE stripe ($A = 0.8$ for the present case). Usually the form of E is very complicated, especially for irregularly-shaped and scattering surfaces.

We apply this calculation method to one of the simplest cases – bending in front of a flat mirror, as schematically illustrated in **Supplementary Figure 6b**. Due to the mirror symmetry, the whole light illumination can be considered as a light cone with its apex at the fiber tip mirror-symmetric position (*LCE'*). The distance between the fiber tip and the light source is 2d, and the entire absorption occurs inside the LCE stripe whose area depends on the bending arc geometry (γ). As can be seen from **Supplementary Figure 6a**, we have relations

$$a = r \cdot sin(\frac{\gamma}{2}) \qquad (5)$$

$$b = 2 \cdot d - r + r \cdot cos(\frac{\gamma}{2}) \qquad (6)$$

$$\bar{d} = d + b/2 \qquad (7)$$

where a is the horizontal distance between LCE edge and the fiber center, b is the vertical distance between LCE edge and the light source and $\bar{d}$ an approximated average distance between the LCE and the light source.

Supplementary Figure 6c shows the schematics of the projection of LCE stripe with respect to the pointed light source on $z = \bar{d}$ plane. The projected area can be approximated as a rectangle, whose length equals to $2a$ and width w_d equals to the LCE width, w_d = 1mm. On $z=\bar{d}$ plane, the reflected light spot has a radius of $R_s = \bar{d} \cdot tan(\beta)$ ($\beta = 13°$ is the divergence angle of light emitted from the fiber tip), and a Gaussian intensity distribution

$$I = \frac{P}{\pi\omega_0^2} e^{-\frac{x^2+y^2}{\omega_0^2}} \qquad (8)$$

where $\omega_0 = \frac{2}{3} R_s$ is the Gaussian beam radius.

For $R_s > a$,

$$E = 2 \cdot A \cdot R \int_l^a I \cdot w_d dy. \qquad (9)$$

For $R_s \leq a$,

$$E = 2 \cdot A \cdot R \int_l^{R_s} I \cdot w_d dy, \qquad (10)$$

where l = 0.5 mm is the half length of the non-actuated region on the LCE stripe (gluing part). Combining Supplementary equations (9) and (10) with equation (3), using R = 0.98 for a flat mirror, and solving Supplementary equation (3), we obtain the central angle γ for different powers P and at different distances d. The results are transferred back to the gripping angle α using Supplementary equation (1), as shown in Supplementary Figure **3b** in the main text.

Note that upon approaching to the closure stage the device reaches a small gripping angle, $\alpha <$ 50°, and the deformed geometry deviates significantly from the arc geometry approximation; at small distances, $d <$ 5 mm, the size of the LCE becomes comparable with the distance, enhancing the inaccuracy in the approximation treatment by using an average distance $\bar{d}$. Thus, there exists a relatively large divergence between the calculated and experimental results at large $|d\alpha|$ and small d regimes.

All numerical calculations are performed with Matlab R2013a.
